# A Comparison of Techniques for Class Imbalance in Deep Learning Classification of Breast Cancer

**DOI:** 10.3390/diagnostics13010067

**Published:** 2022-12-26

**Authors:** Ricky Walsh, Mickael Tardy

**Affiliations:** 1 ISTIC, Campus Beaulieu, Université de Rennes 1, 35700 Rennes, France; 2 Hera-MI SAS, 44800 Saint-Herblain, France; 3 Ecole Centrale Nantes, CNRS, LS2N, UMR 6004, 44000 Nantes, France

**Keywords:** mammography, medical imaging, breast cancer, class imbalance, deep learning, synthetic data

## Abstract

Tools based on deep learning models have been created in recent years to aid radiologists in the diagnosis of breast cancer from mammograms. However, the datasets used to train these models may suffer from class imbalance, i.e., there are often fewer malignant samples than benign or healthy cases, which can bias the model towards the healthy class. In this study, we systematically evaluate several popular techniques to deal with this class imbalance, namely, class weighting, over-sampling, and under-sampling, as well as a synthetic lesion generation approach to increase the number of malignant samples. These techniques are applied when training on three diverse Full-Field Digital Mammography datasets, and tested on in-distribution and out-of-distribution samples. The experiments show that a greater imbalance is associated with a greater bias towards the majority class, which can be counteracted by any of the standard class imbalance techniques. On the other hand, these methods provide no benefit to model performance with respect to Area Under the Curve of the Recall Operating Characteristic (AUC-ROC), and indeed under-sampling leads to a reduction of 0.066 in AUC in the case of a 19:1 benign to malignant imbalance. Our synthetic lesion methodology leads to better performance in most cases, with increases of up to 0.07 in AUC on out-of-distribution test sets over the next best experiment.

## 1. Introduction

Breast cancer is the leading cause of cancer death among women worldwide, and was responsible for an estimated 685,000 deaths in 2020 [1]. Breast screening programs have been introduced in many countries, which, along with early treatment, can significantly reduce mortality rates [2]. These screening programs produce a large number of mammograms, i.e., X-ray images of breasts, requiring the attention of radiologists for diagnosis, which can be a tedious, time-consuming, and costly process [3].

Computer-aided Diagnosis (CADx) tools were proposed to cope with these problems and saw increased use in the early 2000s, albeit with mixed results [4]. More recently, automated diagnosis systems based on Deep Learning (DL) models have demonstrated improved performance and large clinical studies have shown their usefulness as an aid to radiologists in screening mammography [5,6]. These DL models learn to distinguish between benign and malignant cases based on datasets of many retrospective examples.

Datasets used to train these models are often highly imbalanced, i.e., they may contain more benign samples than malignant, as most women who undergo mammography screening do not have breast cancer. This problem of different proportions of each class of interest is called class imbalance, and numerous studies have shown that it can be detrimental to the performance of a classification model [7,8]. This imbalance can cause models to be biased towards the majority class, which is of particular concern in mammography screening as this may lead to models being more likely to predict images as benign, potentially resulting in missed cancers.

Many techniques have been proposed to tackle the effect of class imbalance, but their impact can vary depending on the complexity of the task and the distribution of the dataset [7,8]. In other words, a method that is well suited to optical character recognition, for example, may be unsuitable for cancer classification. This motivates the need for a study of these techniques applied to breast cancer diagnosis using various mammography datasets, to determine if certain techniques are better suited to this task. Moreover, generating synthetic data has shown promise in dealing with this class imbalance problem in mammography in particular [9], but to our knowledge this has not been compared with more common, and less complex, methods.

The aim of this paper can be summarised in the following Research Questions (RQ):

(RQ1) How does an imbalanced training set impact cancer classification performance in mammography?
(RQ2) How do common techniques for tackling class imbalance compare for cancer classification in mammography?
(RQ3) To what extent can synthetic lesions improve classification performance in the presence of class imbalance?

To answer these questions, we conducted experiments on several mammography datasets with different data distributions and levels of class imbalance, including two recently released datasets, Chinese Mammography Database (CMMD) [10] and VinDr-Mammo [11]. The most popular techniques for handling class imbalance were selected from the literature and applied to evaluate their effects on classification performance and generalisability of a standard Convolutional Neural Network (CNN) used for breast cancer classification.

Although this study focuses on mammography, many other medical imaging tasks share the same main characteristics, namely, high-resolution images, a clinical context where the trade-off between sensitivity and specificity is important, and often a high level of imbalance between classes. Thus, the results of this study could have wider applicability for medical imaging in general.

This paper is structured as follows. Section 2 discusses related work on class imbalance. Section 3 details the models, class imbalance techniques, and experiments carried out in this study, with a description of the datasets used in Section 3.3. The results of the experiments are presented in Section 4, which are contextualised and discussed in Section 5. Finally, Section 6 summarises the key findings and future research directions.

## 2. Related Work

### 2.1. Effects of Class Imbalance

Study of the class imbalance problem is not new in the literature. As early as 1993, Anand et al. [12] showed that when training a shallow neural network with backpropagation on an imbalanced dataset, the gradient contribution of the majority class dominates that of the minority class, which leads to slow convergence of the error for the minority class. A significant amount of research has been dedicated to class imbalance in traditional Machine Learning (ML), with a number of workshops and special issues in the early 2000s [13]. The effect has also been demonstrated more recently for CNNs, both by Buda et al. [7] in 2018 in an extensive study on optical character recognition, and by Li et al. [14] in 2021, who showed that a CNN for brain tumor segmentation overfits more on the majority class with a higher level of imbalance.

Several studies have shown class imbalance to be more detrimental for more complex tasks, from earlier findings on traditional ML algorithms [8], and research of multi-layer perceptrons for breast mass classification in 2008 [15], through to the 2018 study of CNNs by Buda et al. [7].

In summary, while the class imbalance problem has been well studied over the past twenty years and its impact has been observed for many domains and datasets, the effect of class imbalance can vary considerably depending on the complexity of the task at hand and the characteristics of the dataset. Moreover, a broad study on class imbalance in automated cancer detection in mammography across several datasets has not been carried out. Although Bria et al. [16] explored similar questions, they used a dataset of patches of 14×14 pixels extracted from the INBreast dataset [17] to classify micro-calcifications, which reveals little about the effect when processing whole images as the task becomes more complex.

### 2.2. Methods for Dealing with Class Imbalance

#### 2.2.1. Common Techniques

Techniques proposed to deal with the class imbalance problem are often categorised as algorithm-level, data-level, or a combination of the two [18]. The most widely used algorithm-based techniques involve changes to the loss function, for example, weighting by the inverse of the class proportions such that the contribution from the majority class and minority class are balanced [18]. This has previously been used for studies in deep learning in mammography [19,20], although Bria et al.found it less effective than over-sampling [16].

The primary techniques in the data-level group include sampling, where either the minority class is over-sampled or the majority class is under-sampled, creating an artificially balanced dataset. There are some drawbacks, in that under-sampling may remove important, informative examples, and over-sampling may lead to overfitting [13]. However, in the context of CNNs, the use of data augmentation during training can reduce the risk of overfitting in general, and so may help to avoid overfitting on over-sampled minority examples. Despite these sampling methods being some of the earliest techniques addressing the class imbalance problem, they remain popular, perhaps due to their simplicity and apparent effectiveness. For example, a review [21] in 2019 of the use of CNNs in mammography discusses the problem of class imbalance and mentions only these two techniques as potential solutions.

In comparing several different sampling strategies, Bria et al. [16] found that over-sampling the malignant class was the most effective, whereas Qu et al. [22] found both over-sampling and under-sampling to be effective in reducing the class imbalance effect for the classification of chest X-rays. Other studies in automated breast cancer classification [23,24] have used an epoch-wise under-sampling strategy, whereby each epoch sees all of the abnormal examples and an equal number of randomly selected normal examples, but this was not compared to other approaches.

The most common methods used to tackle class imbalance in medical imaging studies include the aforementioned over-sampling, under-sampling, and class weighting. However, the effectiveness of these techniques varies depending on the task being performed and the dataset [7,18], and their relative efficacy for classifying whole mammograms remains unclear. Therefore, our study compares the results of applying these common techniques to resolve class imbalance in three heterogeneous datasets of high-resolution mammograms.

#### 2.2.2. Synthesising Images

To avoid overfitting on over-sampled minority examples, further data augmentation can be applied [25], but one step further is to create new synthetic images for the minority class. Several studies have used synthetic data created by Generative Adversarial Networks (GAN) as an additional type of data augmentation during training, alongside typical image flipping, rotation, etc. Realistic low-resolution images created by GANs have helped to improve model performance in studies of classification of liver lesions [26], chest X-ray abnormalities [27], and breast masses [28]. GANs have also shown some success for explicitly tackling class imbalance in mammogram classification [9], although the authors did not compare their approach to other common class imbalance techniques.

The GANs in the above studies were trained to produce low-resolution images up to 256×256 pixels, and so it is unclear how well a GAN would handle whole high-resolution mammograms where lesions can make up less than 1% of the image. One study [29] synthesised higher resolution (i.e., 1280×1024 which is still lower than the original resolution ≈4000×3000) mammograms using a GAN, but required 450,000 images for training, and made no distinction between malignant and benign. Moreover, many studies on GANs dealing with breast lesions require pixel-wise ground truth for lesion locations, which are rarely available in clinical practice.

In this study, we use an alternative method of artificial lesion generation to insert masses, calcifications, and architectural distortions into benign images to tackle class imbalance. Such an approach relates to the augmentation method proposed by Tremblay et al. [30], to cope with a lack of real data through the generation of physically realistic synthetic samples. The mass generation is based on the model of [31], and was previously shown to be useful as a data augmentation technique in training a deep learning model for mass detection [32]. The method we study in this work, including calcifications and distortions, was previously used for segmentation [33], to deal with the absence of ground truth lesion locations.

There are three main advantages of this method compared to the GAN techniques discussed earlier. Firstly, they can be used with images of any resolution, in particular high-resolution images. Secondly, unlike the method of Wu et al. [9], we do not require ground truth lesion locations, which are expensive and rare. Finally, whereas a GAN is confined to the distribution of the dataset at hand, our method of controlling the artificial lesion characteristics allows us to incorporate domain knowledge on the appearance of different lesions, regardless of whether certain lesion types or shapes are represented in the training dataset. Moreover, this method could be used to balance datasets where there is a known shortage of certain types of masses, for example.

In summary, the contributions of the current study are as follows: (i) an analysis of the effect of class imbalance on breast cancer classification using high-resolution images from several recent mammography datasets, (ii) a systematic comparison of the most popular methods for dealing with this class imbalance problem, and (iii) a novel use of synthetically generating abnormalities as an alternative to over-sampling the malignant class.

## 3. Materials and Methods

### 3.1. Dealing with Class Imbalance

We examined several popular methods for addressing class imbalance, namely, (1) class weighting, (2) under-sampling, and (3) over-sampling. We compared these to a synthetic lesion generation technique which we denote here as “artifacting”.

#### 3.1.1. Class Weighting

One of the simplest approaches is to apply a higher weight to the minority class during training when calculating the loss. Equation (1) below shows how the weights were calculated. We used global weights, i.e., using the total numbers of minority and majority samples in the training dataset rather than, for example, calculating the ratio for each mini-batch during training. This decision was based on our small batch size of 8 images relative to the large class imbalance of 19:1 in the VinDR dataset, i.e., 19 benign images for every malignant case (see Section 3.3). Thus, calculating class weights for each batch would mean the largest weight any minority sample could possibly receive would be 7, which would not balance the loss contributions of both classes.

(1) $$w_{\mathrm{minority}}=\frac{\#\;\mathrm{of}\;\mathrm{Majority}\;\mathrm{Samples}}{\#\;\mathrm{of}\;\mathrm{Minority}\;\mathrm{Samples}},\;w_{\mathrm{majority}}=1$$

#### 3.1.2. Under-Sampling

With this approach, a fixed random sample of the majority class is taken before training so that the number of minority and majority examples are balanced. For datasets with large imbalance between classes, this leads to a dramatic reduction of the training data size, e.g., a 90% reduction of the training data size for the VinDR dataset, which has a benign to malignant ratio of 19:1. For cases where the class imbalance is less severe, like our two other datasets, under-sampling removes fewer samples and thus less potentially valuable information is lost.

#### 3.1.3. Over-Sampling

For this method, each majority class example is seen exactly once in every epoch whereas minority class examples can be seen multiple times. More specifically, we first fill half of the batch with unseen majority examples and then randomly select minority examples to complete the rest of the batch. The epoch is complete when all majority examples have been seen.

#### 3.1.4. Artifacting

The final method, and a contribution of this study, involved inserting synthetic malignant lesions [33] into benign images during training to balance the benign and malignant classes. We used this method only for datasets where there are more benign samples than malignant (i.e., VinDR and HMI, see Section 3.3), as if a malignant lesion is inserted into a benign image it can be considered malignant, but inserting a benign lesion into a malignant image does not change the class of the image, as the source of malignancy would still be there.

**Figure 1:**
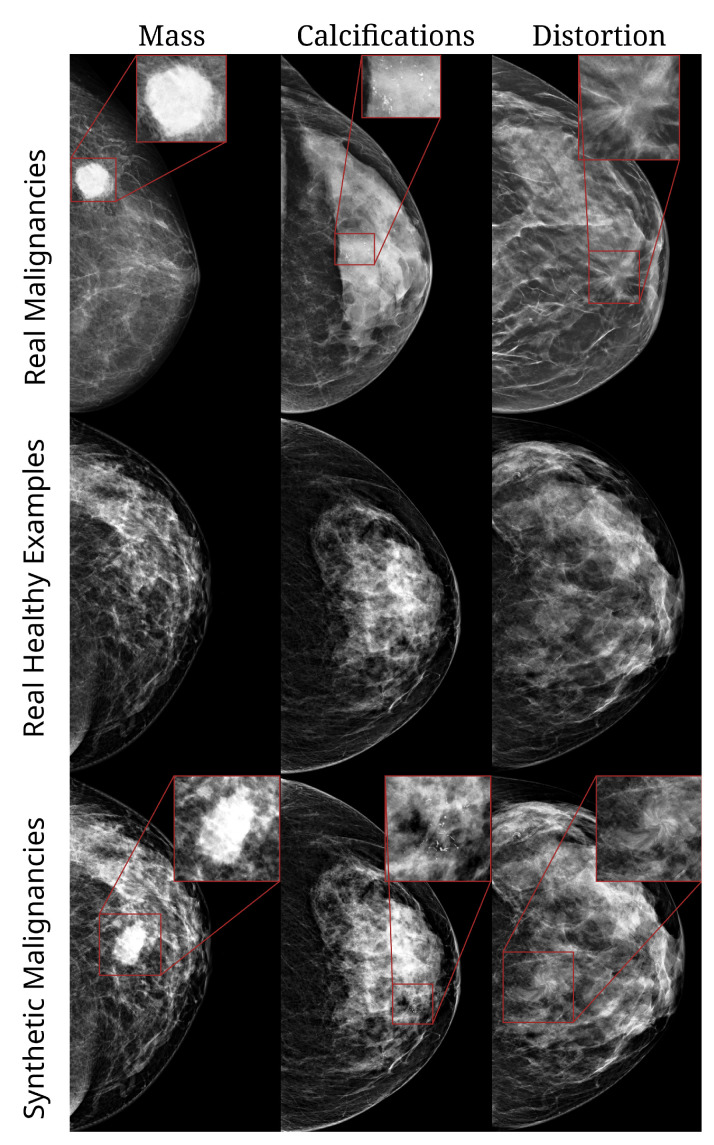
Examples of images from HMI dataset. **Top row**: real malignant examples of a mass, cluster of calcifications, and architectural distortion, respectively. **Second row**: healthy/normal breasts. **Third row**: healthy breasts with synthetic lesions (mass, calcifications, and distortion, respectively).

Three types of malignant lesions were inserted, namely, masses, calcifications, and architectural distortions, examples of which are shown in Figure 1. The methodology underlying the generation of these synthetic lesions has been described in detail in [34] and has previously been employed for lesion segmentation [33]. We will give a brief description here.

The mass generation is based on an independent implementation [35] of the computational model designed by de Sisternes et al. [31]. This method uses a stochastic Gaussian random sphere model to generate synthetic masses and then adds spicules to these masses with an iterative branching algorithm. The authors showed that both radiologists and CAD tools had difficulty in separating real masses from synthetic samples.

A calcification is an accumulation of hardened calcium in the breast tissue. While most calcifications seen in mammograms are benign, some types of calcification are more indicative of malignancy such as smaller calcifications and clusters [36]. Therefore, similarly to [33], we imitated these malignant clusters by inserting localised regions of small bright spots where each calcification has a diameter of between 0.25 and 1 mm, formed in convex groups of high intensity pixels.

An architectural distortion, which can be defined as a distortion of the breast parenchymal architecture without a definable mass [37], is another type of finding which can be associated with malignancy. We considered the case where these manifest in a mammogram as a twisting or compression of the tissue in a localised region. We created artifacts with a local non-linear geometric transformation using the swirl function from the scikit-image [38] Python library, which allowed us to approximate such distortion effects.

These three types of synthetic lesions were inserted in a randomised fashion during training. Firstly, each training mini-batch of eight images was half-filled with benign samples. Then, two real malignant samples were added, and the remaining two places were filled by randomly selecting two of the benign samples in the batch and inserting 1, 2, or 3 synthetic lesions. This means that for $D_{{\mathrm{VinDR}}_{\mathrm{train}}}$, which has a benign to malignant ratio of 19:1, we are both over-sampling the real malignant examples and using synthetic malignant examples. Ensuring that there are both real and synthetic examples in each batch was found experimentally to yield the best results.

### 3.2. Experimental Framework

The experimental methodology involved applying each of the above techniques for tackling class imbalance to each dataset while training a deep learning model for classification.

#### 3.2.1. Pre-Processing

The images were pre-processed in an automated fashion before being used for training or testing a classification model. The first step was to remove the background noise using the Triangle threshold method [39]. Next, to detect the breast region, we binarised the cleaned image and we computed connected components with scikit-image [38] in Python. The breast was assumed to be the largest connected component, allowing us to remove smaller regions corresponding to labels and markings by setting their pixel values to zero. Images of right breasts were flipped so that all breasts were located at the left side of the images. The height was cropped to the size of the breast, and re-sized to 2048 pixels. The aspect ratio was maintained between the height and width, but the right side of the image was padded with zero-intensity pixels to give a square 2048×2048 image. Finally, we applied histogram normalisation to the pixels’ intensities, enhancing the contrast of the images, and we scaled the values to the range ∈[0,1].

#### 3.2.2. Training

A ResNet-22 architecture, which has previously been applied to breast cancer classification [23] was adopted as the deep learning classifier. The same version was used in this study as in the encoder block of [40], with five residual blocks and an increasing number of filters (16, 32, 64, 128, 256), and using separable convolutions and instance normalisation as in [40]. During training, the images were augmented in a randomised fashion, including vertical flipping and translation (<100 pixels), rotation (±10 degrees), and zooming, as well as inpainting random patches in the image similar to [41].

For each experiment, the model was trained from scratch for 100 epochs with a categorical cross-entropy loss, using the Adam optimiser with a learning rate of $5\times 10^{-4}$, informed by previous experiments on the same data and a small number of tests during this study. The batch size was set to 8, which was the maximum possible without exceeding available memory on the NVIDIA RTX 2080 Ti GPU used in this work. The training times of the 14 experiments varied between 1 and 8 days each, so extensive hyper-parameter tuning was not feasible within a reasonable period of time, and thus was out of scope of the present work.

During training, the Area Under the Curve of the Receiver Operating Characteristic (AUC-ROC) was calculated on the validation set (see details on data splits in Section 3.3), and the five models that achieved the best AUC were selected. Metrics on the test sets were ultimately calculated for each of these five models, and the mean score for each experiment is reported in Section 4.

#### 3.2.3. Evaluation

When evaluating predictions on the test set, we assessed the breast-wise predictions, similar to [42]. For many of the patients in our datasets, there were two images of each breast, one from each of the Craniocaudal (CC) and Medio-lateral Oblique (MLO) views. Signs of malignancy might appear on only one of these views, so we combined the predictions at test-time by taking the average of the model’s predicted probability of malignancy for each breast.

We evaluated the performance of each trained model on a test set from the same distribution as the training set, as well as on the other out-of-distribution test sets, exploring the generalisation capabilities of each method. We used AUC-ROC as our primary evaluation metric. ROC is a plot of Sensitivity (Se) vs. (1-Specificity (Sp)) (see Equation (2)) for many operating points of the model, i.e., thresholds on the softmax outputs used to decide whether to classify an example as malignant or benign. AUC-ROC captures the information in this curve in one single metric, allowing the overall quality of the model to be evaluated without choosing a specific threshold. One of the benefits of this for the current study is that setting a different threshold for a trained model is itself a technique for tackling class imbalance [7,18], achieving a different balance between sensitivity and specificity. Therefore, assessing the overall model performance is more informative here than performance at one single threshold.

There has been some criticism of AUC-ROC for classification of imbalanced classes, in favour of the alternative Area Under the Precision-Recall Curve (AUC-PR) [43,44]. On the other hand, some studies [45,46] have found AUC-ROC to perform well under class imbalance. The main reason to use AUC-ROC here is that it is commonly used in studies of breast cancer classification and segmentation [19,23,40,47], allowing comparisons to other works.

We also assessed predictions at a standard operating point of 0.5. We calculated Sensitivity (Se) and Specificity (Sp) (see Equation (2)) to evaluate how biased each trained model was towards malignant or benign samples.

(2) $$Se=\frac{TP}{TP+FN}\;\;\;Sp=\frac{TN}{TN+FP}$$

We calculated Matthews Correlation Coefficient (MCC) (see Equation (3)) to have a single evaluation metric which incorporates information from True Positives (TP), True Negatives (TN), False Positives (FP), and False Negatives (FN). Several studies [46,48] have shown MCC to be an informative metric in the study of class imbalance problems. Finally, to get an estimate of the standard error of the metrics for each experiment, we used bootstrapping [49] with 1000 samples.

(3) $$MCC=\frac{TP\cdot TN-FP\cdot FN}{\sqrt{\left(TP+FP)\cdot (TP+FN)\cdot (TN+FP)\cdot (TN+FN\right)}}$$

### 3.3. Data and Labels

The task we consider is to classify samples between malignant or non-malignant (including normal cases and benign lesions). In most cases, we used the Breast Imaging-Reporting and Data System (BI-RADS) risk rating from the American College of Radiology (ACR) [50] to determine malignancy, where the scores are categorised as follows: (1) negative or normal, (2) benign, (3) probably benign, (4) suspicious for malignancy, (5) highly suggestive of malignancy, and (6) known biopsy-proven malignancy. We binarised the rating for each image taking scores 1, 2, and 3 as non-malignant, and BI-RADS 4, 5, and 6 as malignant. In the case of the CMMD dataset these ratings are not available but each breast has been confirmed by biopsy to be benign or malignant, so we used these binary labels.

We used several heterogeneous public datasets, and a private dataset, each based on different geographical locations and with different levels of class imbalance, to assess the performance of class imbalance techniques. Each of the datasets contains high-resolution Full Field Digital Mammography (FFDM) images. This reflects current clinical practice, and is the reason we chose not to use the popular Digital Database for Screening Mammography (DDSM) dataset, given that those images are scanned and digitised Screen-Film Mammograms. A full breakdown of the number of images in each dataset is given in Table 1.

The first dataset used for training was a private dataset of 3847 images, containing mammograms from four vendors, namely, Fujifilm, GE, Hologic, and Planmed. Benign images account for 70% of the training set. We used a test split for this dataset containing 504 images selected randomly. We refer to the datasets as $D_{{\mathrm{HMI}}_{\mathrm{train}}}$ and $D_{{\mathrm{HMI}}_{\mathrm{test}}}$.

The first of the public datasets used for training was VinDr-Mammo [11], consisting of 20,000 images from 5000 patients. The images were collected in two hospitals in Vietnam, Hanoi Medical University Hospital and Hospital 108, using mammography systems from three vendors (Siemens, Planmed, and Giotto). This dataset presents a large class imbalance, where only 988 (5%) images are labelled as malignant, being close to a typical mammography screening scenario where most of the images acquired are normal or benign. The dataset authors have already split the data into training (16,000) and test (4000) images to ensure consistency in results reported. Hereafter, these will be referred to as $D_{{\mathrm{VinDR}}_{\mathrm{train}}}$ and $D_{{\mathrm{VinDR}}_{\mathrm{test}}}$, respectively.

The second public dataset used for training was the relatively new Chinese Mammography Database (CMMD) [10], which contains 3744 images of 1775 patients. The dataset was published by the South China University of Technology, and the images were obtained using a GE Senographe DS mammography system. In this dataset there are more malignant images than benign cases, reflecting the diagnostic clinical context, where suspicious findings have been identified and further imaging is required to diagnose if they are malignant. We split the dataset into training (2998 images—80%) and test (746 images—20%) sets, stratifying by class and ensuring that the images for a single patient remained in a single set. We refer to these datasets as $D_{{\mathrm{CMMD}}_{\mathrm{train}}}$ and $D_{{\mathrm{CMMD}}_{\mathrm{test}}}$.

For each of the datasets, we further divided the training set into separate training and validation sets, using the validation set to tune hyper-parameters and to find the epoch of the “best” model during training. These splits were stratified based on class, and images for each patient appeared in only one group.

For each assessed technique tackling class imbalance, the models were separately trained on each of the three above datasets, and the performance was assessed on each of the test sets. Taking a model trained on $D_{{\mathrm{VinDR}}_{\mathrm{train}}}$ and testing it on $D_{{\mathrm{CMMD}}_{\mathrm{test}}}$, for example, allows us to assess the generalisation capability on datasets not seen at training time.

Finally, the popular public INBreast [17] dataset was used solely for testing performance across all experiments, allowing a comparison to other studies in the area [42]. This dataset consists of 410 FFDM images taken with a Siemens mammography system.

### 3.4. Software

All of the image processing and experiments in this study were carried out using Python [51]. Image processing was done using functions developed by the Hera-MI team on top of the OpenCV [52] and scikit-image [38] packages. Keras [53] and Tensorflow [54] were used to build and train the classification model. The Matplotlib [55] and Seaborn [56] packages were used for plotting.

## 4. Results

In this section, we first report overall performance of each experiment on all test sets in Section 4.1, based on the breast-wise AUC-ROC scores in Table 2. Secondly, in Section 4.2 we analyse the sensitivity and specificity resulting from the various treatments.

### 4.1. Breast-Wise AUC Performance

When training on $D_{{\mathrm{HMI}}_{\mathrm{train}}}$, and testing on the corresponding test set $D_{{\mathrm{HMI}}_{\mathrm{test}}}$, we do not observe significant differences between simply training on the imbalanced dataset (AUC=0.776) and applying standard class imbalance techniques. Whereas the class weighting leads to a slight increase in AUC (0.786), models trained with over-sampling see a decrease of 0.014 to 0.762. There is a wider range of results when considering out-of-distribution generalisation. Of these four initial experiments, the imbalanced training leads to the best performance on $D_{{\mathrm{VinDR}}_{\mathrm{test}}}$ and INBreast, whereas the models trained with class weighting perform slightly better on $D_{{\mathrm{CMMD}}_{\mathrm{test}}}$ (0.660 vs. 0.646), although the generalisation to this dataset remains quite poor.

The models trained on $D_{{\mathrm{HMI}}_{\mathrm{train}}}$ using our synthetic lesions (*Artifacted*) method achieve the best results on the related $D_{{\mathrm{HMI}}_{\mathrm{test}}}$, with an improvement of Δ=0.021 over the next best AUC score. Moreover, this method achieves a noticeable increase in generalisability to out-of-distribution test sets, with improvements over the next best result for $D_{{\mathrm{VinDR}}_{\mathrm{test}}}$ (Δ=0.060), $D_{{\mathrm{CMMD}}_{\mathrm{test}}}$ (Δ=0.070), and INBreast (Δ=0.027). Most notably, this generalisability results in the same performance on out-of-distribution datasets as when training directly on images from those distributions ($D_{{\mathrm{VinDR}}_{\mathrm{test}}}$: 0.760 vs. 0.757, $D_{{\mathrm{CMMD}}_{\mathrm{test}}}$: 0.730 vs. 0.727).

Turning our attention to the models trained on $D_{{\mathrm{VinDR}}_{\mathrm{train}}}$, we see minor drops in the AUC scores for $D_{{\mathrm{VinDR}}_{\mathrm{test}}}$ when comparing the imbalanced training (0.757) to over-sampling (0.752), and class weighting (0.739). However, a more significant decrease occurs for the under-sampling experiment (0.691, Δ=−0.066), when we remove a large portion of the benign samples from the dataset. The over-sampled model achieves the best results on INBreast (0.824), which is the second highest result on INBreast among all experiments, perhaps due to the high representation of Siemens images in $D_{{\mathrm{VinDR}}_{\mathrm{train}}}$. The models trained on $D_{{\mathrm{VinDR}}_{\mathrm{train}}}$ deliver relatively poor generalisation to both $D_{{\mathrm{HMI}}_{\mathrm{test}}}$ and $D_{{\mathrm{CMMD}}_{\mathrm{test}}}$, again due to the characteristics of images generated by different mammography systems, as well as an insufficient number and variety of malignant samples in $D_{{\mathrm{VinDR}}_{\mathrm{train}}}$.

The *Artifacted* method, trained on $D_{{\mathrm{VinDR}}_{\mathrm{train}}}$, demonstrates an improvement in AUC over the next best method on $D_{{\mathrm{VinDR}}_{\mathrm{test}}}$, i.e., the imbalanced training (Δ=0.011), as well as better generalisability to $D_{{\mathrm{HMI}}_{\mathrm{test}}}$ (Δ=0.040). It also yields the second best AUC score on INBreast (0.799) among the five results in this second set of experiments. In contrast, this method delivers the lowest result for $D_{{\mathrm{CMMD}}_{\mathrm{test}}}$, with a difference of Δ=−0.041 compared with class weighting.

The models in the final set of experiments are trained on $D_{{\mathrm{CMMD}}_{\mathrm{train}}}$, and here we do not have an *Artifacted* experiment as the nature of the imbalance is different in the CMMD dataset, i.e., there are more malignant samples than benign (see Table 1). The imbalanced (0.727) and over-sampled (0.719) experiments offer similar AUC performance when tested on $D_{{\mathrm{CMMD}}_{\mathrm{test}}}$, with a gap over the next two results, under-sampled (0.690) and class weighting (0.681). Most of the trained models perform very poorly when applied to $D_{{\mathrm{HMI}}_{\mathrm{test}}}$ and $D_{{\mathrm{VinDR}}_{\mathrm{test}}}$, with AUC scores close to 0.5, which would be achieved by a predictor making random guesses. The over-sampling experiment is an exception to this with the highest AUC scores for $D_{{\mathrm{HMI}}_{\mathrm{test}}}$ (0.614), $D_{{\mathrm{VinDR}}_{\mathrm{test}}}$ (0.667), and INBreast (0.711). These results are still poor, however, and indeed the CMMD dataset is associated with the lowest results across all experiments, both on the in-distribution test set, and the ability of a model trained on $D_{{\mathrm{CMMD}}_{\mathrm{train}}}$ to generalise to any of the other three datasets.

### 4.2. Studying Sensitivity, Specificity, and MCC

The first observation coming from the results reported in Table 3 is that an imbalance in the training data is reflected in imbalances between sensitivity and specificity of the trained models. In total, 70% of training samples in $D_{\mathrm{HMI}}$ are benign, resulting in a lower sensitivity (Se) than specificity (Sp) in the trained model (Se=0.574, Sp=0.824). Similarly, 70% of the images in $D_{\mathrm{CMMD}}$ are malignant resulting in a higher sensitivity (Se=0.781, Sp=0.479). The most severe example of this phenomenon is visible with $D_{\mathrm{VinDR}}$, where benign samples outnumber malignant 19 to 1, and as a result nearly all test samples are classified as benign by the model (Se=0.004, Sp=1.0). In general, the techniques employed to tackle class imbalance also bring sensitivity and specificity values closer to each other. The only exceptions to this were the application of class weighting on $D_{\mathrm{HMI}}$, where sensitivity reduced from the imbalanced case, and over-sampling with $D_{\mathrm{CMMD}}$, which saw a decrease in specificity.

Despite the gap between sensitivity and specificity when training on the imbalanced datasets, the results for MCC are in fact higher than when applying the three standard class imbalance techniques (under-sampling, over-sampling, and class weighting) for both $D_{\mathrm{HMI}}$ and $D_{\mathrm{CMMD}}$. However, this observation does not hold in extreme cases when the class imbalance is more severe as for $D_{\mathrm{VinDR}}$, where the near-zero sensitivity contributes to a very low MCC of 0.033.

The *Artifacted* method achieves the highest MCC score for $D_{\mathrm{HMI}}$ (MCC=0.443), as well as the second best result for $D_{\mathrm{VinDR}}$ (0.253). The highest result for the latter case is given by the over-sampled experiment (0.378); however, this coincides with a low sensitivity (0.263), which indicates that the model may have difficulty generalising to malignant samples different from those in the training set.

**Figure 2:**
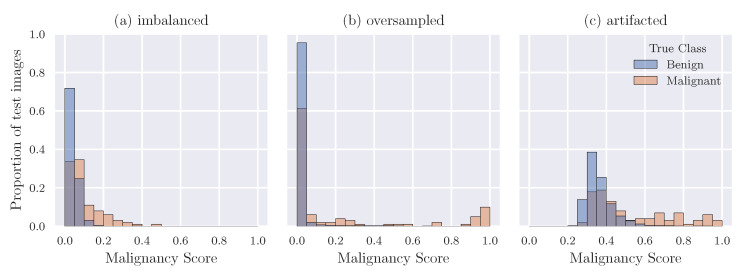
Distributions of predictions for selected models, trained on $D_{{\mathrm{VinDR}}_{\mathrm{train}}}$ and applied to $D_{{\mathrm{VinDR}}_{\mathrm{test}}}$. Malignancy score is the softmax output of the model—at an operating point of 0.5, for example, the model would predict malignant for samples above 0.5, and benign for those below. The distributions in the plot are normalised so that bar heights sum to 1 for each class in each plot.

Figure 2 demonstrates the behaviour of the models trained on $D_{{\mathrm{VinDR}}_{\mathrm{train}}}$ with different approaches. With an imbalanced dataset, the model has a strong bias towards the benign class, meaning that almost every sample is given a low probability of malignancy at test-time, resulting in the low sensitivity and high specificity seen at an operating point of 0.5. However, the trained model remains capable of achieving some separation between malignant and benign samples, leading to the second highest AUC score among the VinDR experiments. For example, over 70% of truly benign samples are given a malignancy score between 0 and 0.05, whereas fewer than 40% of truly malignant cases are placed in this bracket.

Over-sampling resolves the problem of every sample being classified as benign. However, the resulting behaviour remains unusual and undesirable. Nearly 100% of normal or benign breasts, as well as over 60% of breasts with malignant lesions, are assigned a low score of malignancy between 0 and 0.05 by the model. Conversely, almost 20% of malignant samples attain a high malignancy score between 0.9 and 1. This behaviour may indicate overfitting on the small number of malignant samples in the training set, leading the model to correctly predict malignancy with high confidence for samples who share some characteristics with those in the training set, while predicting most samples in the test set to be benign with high confidence.

The *Artifacted* model yields a more natural distribution of predictions on the test set in that the range is more spread, allowing us to separate what might be considered high confidence from low confidence. However, two problems can be observed in Figure 2c. Firstly, a large proportion of truly malignant breasts still attain predicted malignancy scores similar to those assigned to benign samples, particularly in the range [0.3,0.45]. Secondly, virtually zero samples are assigned a malignancy score less than 0.25. The exact reason for this behaviour is yet to be determined, but potential causes are discussed in Section 5.

## 5. Discussion

### 5.1. Research Questions

In this section, we refer to the three Research Questions (RQ) listed in the introduction (see Section 1) and discuss the insights with regard to each of these questions.

(RQ1) How does an imbalanced training set impact cancer classification performance in mammography?

For each of the three imbalanced datasets, the class imbalance caused the classifier to bias towards the majority class. As might be expected, a higher imbalance resulted in a higher bias, i.e., a standard classifier trained on the VinDR dataset predicted every test sample to be benign. On the other hand, models trained on imbalanced datasets unexpectedly achieved comparable AUC-ROC scores to models trained with common class imbalance techniques, indicating that despite predictions shifting towards the majority class, the model still learns to separate the two classes. This lends credence to setting a new threshold on the output scores post-training as an effective technique of dealing with class imbalance, applied in [7] (see also [18]). New thresholds can also be set in practice, regardless of class imbalance, to achieve an acceptable trade-off between sensitivity and specificity for a given task. Therefore, threshold setting may be the simplest approach to tackling class imbalance, while remaining relatively effective.

(RQ2) How do common techniques for tackling class imbalance compare for cancer classification in mammography?

Among over-sampling, under-sampling, and class weighting, no single technique consistently achieved the best AUC-ROC nor MCC scores across all experiments. Over-sampling performed best of these techniques on the VinDR and CMMD experiments, in the latter case also leading to a significant improvement when testing on out-of-distribution datasets. However, over-sampling performed the worst on three out of four test sets when trained on HMI. Therefore, there is clearly no one single best approach for every mammography dataset; however, one lesson that may be gained from these results is that experimenting with other techniques may allow for an improved out-of-distribution generalisation without sacrificing in-distribution performance.

There is a notable drop in classification performance in the VinDR and CMMD experiments when under-sampling is applied. This aligns with expectations that using only a subset of data may remove informative samples and thus may hinder performance, particularly in the case of VinDR where 90% of the dataset is removed. Furthermore, the selection of the subset for training affects performance, as the model began to learn only after several attempts with different random seeds for the initialisation of model weights and taking a different random sample of benign images. The training process was not straightforward either for the imbalanced or class weighting experiments on the VinDR dataset. We hypothesise that these difficulties might be due to the low frequency with which malignant samples were seen during training. In our case, training loss began to decrease after we ensured that approximately an equal number of malignant samples appeared every 0.2 epochs, although it remains unclear whether this was the direct cause.

Over-sampling, together with the *Artifacted* method, were the only VinDR experiments that worked without further adjustments. Moreover, the model trained on VinDR with over-sampling performed well on the test set relative to the other methods according to our AUC-ROC and MCC metrics (see Section 4), so over-sampling could be seen as a promising approach for cases with high imbalance between classes. On the other hand, the distribution of predictions of this model showed signs of overfitting on the small number of minority samples in the training set, which is a common concern of using over-sampling. This output distribution may be problematic in cases where the softmax outputs are used to gain information about the prediction uncertainty, e.g., in [57]. Future research is needed to determine whether this behaviour can be resolved, for example, by further data augmentation methods beyond those used in this work (see Section 3.2.2), or a combined over-sampling/under-sampling approach as in [7].

(RQ3) To what extent can synthetic lesions improve classification performance in the presence of class imbalance?

The results showed that inserting synthetic lesions into benign samples during training can be a useful technique to balance classes. When applied to the HMI data, our *Artifacted* achieves an improvement of 0.021 in ROC-AUC over the next best method, as well as significant improvements in ROC-AUC on the out-of-distribution test sets, up to Δ=0.07. Applied to the more highly imbalanced data, VinDR, the performance improvement is more modest (Δ=0.011), and the out-of-distribution performance is mixed. Despite these mixed results, the *Artifacted* model shows more promise as it does not suffer from the overfitting behaviour demonstrated by the model trained with over-sampling.

The distribution of output predictions for the *Artifacted* model (Figure 2c) shows that no samples were given a malignancy score lower than 0.25 (where if a sample receives a low score, the model is more confident it is benign). The cause of this is unclear, but perhaps either noisy labels during training, or difficulty in distinguishing malignant lesions from benign lesions may lead to this behaviour. Lesions appear in only one of the two views (CC or MLO) for approximately 10% of malignant breasts in the VinDR dataset, so the other image will be included as malignant during training despite containing no visible signs of malignancy. Predicting these images to be benign during training would lead to a high contribution to the loss, perhaps causing the model to generally avoid assigning a low malignancy score to images.

**Figure 3:**
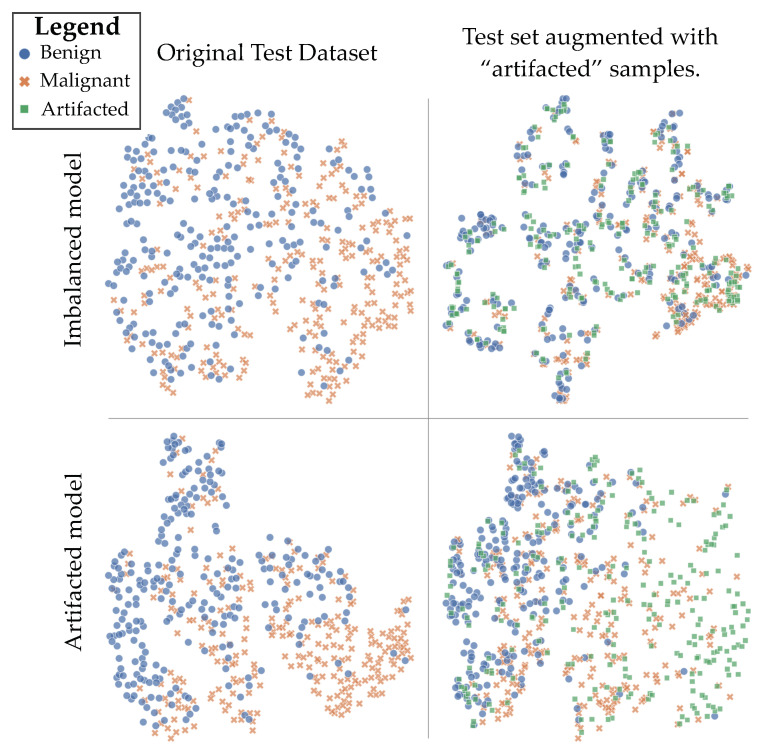
Examining $D_{{\mathrm{HMI}}_{\mathrm{test}}}$ in the latent space of models trained on $D_{{\mathrm{HMI}}_{\mathrm{train}}}$. Shown is the output of t-SNE dimensionality reduction. **Top row**: imbalanced model; **bottom row**: “artifacted” model; **left column**: original test dataset, **right column**: test set augmented with “artifacted” samples.

We study the effect of “artifacting” observing the latent representation of the test samples from the HMI dataset, as well as when we insert synthetic lesions into these images (see Figure 3). When we introduce “artifacted” samples to a model that has not seen them at training time (top-right pane), multiple tight clusters are formed in the latent space, and the representation of these synthesised examples is closely related to real examples in the test set, both benign and malignant. When the model has encountered “artifacted” samples during training, a broader latent space representation appears, and some of the synthesised examples occupy their own regions of the space (bottom-right pane). This indicates that some of the synthetic lesions are quite different to the real lesions in the HMI dataset. This helps the model to learn from a broader data distribution and helps to explain the significant increases in out-of-distribution performance seen by the *Artifacted* method. A further illustration of this effect is seen by comparing the top-left and bottom-left panes, where the addition of synthesised malignant images during training has the effect of pushing the real malignant images closer together in the latent space.

### 5.2. Comparison to Previous Work

Our findings both support and disagree with some of the conclusions of Buda et al. [7] who carried out a systematic review of the behaviour of CNNs for digit recognition under class imbalance. That study found that over-sampling generally performed best in terms of AUC-ROC results, but the performance diverged from the baseline only as the number of minority classes and the imbalance ratio increased. In our case, we have only one minority class, and smaller imbalance ratios (at most 19:1) than many of the situations they examined, so this aligns with our results showing that most standard techniques dealing with class imbalance gave no significant benefit to AUC-ROC over the baseline. The authors also claimed that over-sampling with CNNs did not cause overfitting. However, the false overconfidence of our model trained on the VinDR dataset with over-sampling indicates that overfitting may still remain a problem.

Stadnick et al. [42] tested several state-of-the-art models [19,23,24,58] on multiple public datasets, including INBreast and CMMD. The authors used only 26% of the INBreast images in their test set, and test on the full CMMD dataset, so we re-evaluated our *Artifacted* model trained on $D_{{\mathrm{HMI}}_{\mathrm{train}}}$, yielding an AUC-ROC of 0.850 on this INBreast subset and 0.718 on the full labelled CMMD dataset of 3728 images. The AUC-ROC scores reported by [42] vary between 0.612–0.980 for INBreast and 0.449–0.831 for CMMD, and our results outperform three of the seven reported methods on INBreast, and two on CMMD.

Our results are most similar to the model of Wu et al. [23], which scores 0.802 on INBreast and 0.740 on CMMD. Remarkably, Wu et al. [23] rely on a significantly larger dataset with both a higher amount of malignant samples and a substantially larger imbalance. On the other hand, we note that the dataset is composed of images coming from four different vendors, similar to our HMI dataset. This leads us to two observations: firstly, the amount of malignant samples is more likely to determine the model performance and the “artifacting” method, used in this work, allows us to cope with insufficient data; secondly, the dataset heterogeneity can positively influence the performance of the training.

The VinDR and CMMD datasets are both relatively new, and so are not as well studied as INBreast or DDSM, for example. Thus, our work provides a useful reference and baseline for other researchers using these datasets. We note the lower results achieved on the CMMD dataset, both in this study and in [42], and one potential cause of this is the composition of the dataset. All breasts in the CMMD dataset have been biopsied, indicating that there are suspicious abnormalities in both the malignant and benign images, making the task of separating malignant from non-malignant more difficult. Using a different dataset, Wu et al. [23] found lower classification performance on a biopsied sub-population compared to their overall screening population.

A goal of this paper was to fairly compare popular class imbalance techniques using several datasets and consistent processing pipelines and metrics. Too often, new techniques are proposed for coping with class imbalance, or for generally improving breast cancer classification and segmentation, where the studies provide no comparison to existing methods or they compare methods using proprietary datasets or specific, non-standard performance metrics. For example, while the AUC-ROC metric is generally used in research, studying specific operating points is more relevant from an industrial application perspective. Further standardisation of evaluation and performance measurement will help researchers in the field to compare the methods from the large body of research in this field and more efficiently find techniques applicable to their problems, and the current work as well as other comparative studies such as [42] help in this regard. Access to public datasets is also crucial for these purposes, so the release of two FFDM datasets (VinDr-Mammo [11] and CMMD [10]) in the last two years is very welcome.

### 5.3. Implications and Recommendations

Why is class imbalance detrimental? Because it may bias the trained models towards predicting the majority class. Our results show that in some cases, despite this shift in output prediction scores, the model may still be capable of discriminating between samples from different classes to the same extent as when typical class imbalance techniques are applied, as evidenced by the similar AUC-ROC scores. Thus, if the imbalance ratio is not extreme, setting a (data-specific) threshold on the softmax outputs should suffice to achieve the desired trade-off between sensitivity and specificity. On the other hand, if the context necessitates more realistic prediction scores, indicative of the confidence of the predictions for individual samples, then applying a technique for dealing with this imbalance will be important.

For cases with a higher class imbalance, our experiment with VinDR indicates that over-sampling may yield the best separation between malignant and benign, but the output distributions of the predictions should be assessed to ensure the model does not overfit on the small number of minority samples. Finally, if feasible within the context of the study, whether on mammography or medical imaging more broadly, generating synthetic lesions could provide a good way of balancing the classes while also introducing prior knowledge from domain experts, not only reducing the bias towards the benign class, but also improving the overall model performance.

Overall, our experiments tend to demonstrate that better performance is achieved when training on more heterogeneous and augmented data. The best results are achieved on the HMI multi-vendor (five vendors) dataset with synthesised lesions. VinDR is globally a runner-up with three vendors included. Finally, CMMD-trained classifiers perform the worst having only one vendor in the dataset.

Certification of deep learning solutions for breast cancer detection is usually based on demonstrating efficacy on a particular dataset collected by the vendor. It is well-known in the machine learning community that performance may decrease if the distribution of data used for training is different to the cases on which the model is applied (known as a “domain shift”) [59]. The results in this study provide further evidence of this, for example, with models trained on CMMD data failing to generalise to the other datasets. The regulatory process ought to account for this performance degradation under the presence of dataset shift, requiring systems to demonstrate safety and efficacy on sample images from all “domains” (in this case, mammography imaging systems) where the deep learning solution will be applied. The *Artifacted* method employed in this study could then help to improve generalisation to different data distributions, requiring fewer annotated malignant samples to be collected from those domains. This is particularly appealing in the context of data protection policies such as General Data Protection Regulation (GDPR) and Health Insurance Portability and Accountability Act (HIPAA). Indeed, malignant data samples are more likely to reveal patient identity because the number of patients with a specific form of cancer is much lower than the general, healthy population, hence reducing the amount of real malignant samples used reduces the risk of privacy leaks.

### 5.4. Limitations

Due to the large number of experiments, and the time for each training run (1–8 days), it was not feasible to conduct extensive hyper-parameter tuning. While the application of techniques such as Batch Normalisation or Dropout have been shown to increase robustness with respect to hyper-parameters, better results may have been possible for each individual experiment with more in-depth investigation. In our case, for the sake of simplicity and a fair comparison, we chose to use the same setup (learning rate, number of epochs, etc.) for all experiments. We focused on studying class imbalance in the context of deep-learning-based classifiers, reducing the complexity of the studied neural networks. More advanced solutions may yield different results on the same datasets.

There are many proposed techniques for tackling class imbalance, and only a subset of the most commonly used are considered here. However, it is possible that other, more complex methods may improve results similar to Synthetic Minority Over-sampling Technique (SMOTE) [60] improving performance for traditional machine learning problems. More complex methods are, by their nature, more difficult to implement and tune. Hence, it may take time for a single technique to become more popular than simpler sampling and weighting strategies. Until such time as one of the many techniques available proves superior for many domains and tasks, our study will provide useful insights to researchers in mammography, and medical imaging more broadly.

## 6. Conclusions

This study reviews several popular class imbalance techniques as applied to breast cancer classification in high-resolution mammography using the latest public datasets. The results show that a higher class imbalance when training a deep learning classifier causes a greater bias towards the majority class, which is generally reduced by applying any of the standard class imbalance techniques. However, these techniques often provide no benefit to overall model quality as measured by AUC-ROC, indicating that setting a new threshold or operating point after training may well be sufficient in many use cases. Moreover, under-sampling in particular led to a significant drop in AUC-ROC in cases of higher imbalance between classes. Furthermore, the study demonstrates the usefulness of synthetic malignant lesions to balance classes, both in eliminating the bias towards the majority class, and in achieving higher AUC-ROC performance, with some significant improvements to out-of-distribution generalisation. Future research could explore using synthetic lesions in a more targeted way to increase the number of examples of lesion types under-represented in the dataset.

## Figures and Tables

**Table 1:** Summary of the number of images in each dataset and subset by class.

Dataset	Training		Validation		Test		Total
	Normal/Benign	Malignant	Normal/Benign	Malignant	Normal/Benign	Malignant	
HMI	1952 (70%)	856 (30%)	402 (75%)	133 (25%)	257 (51%)	247 (49%)	3847
VinDR	13,286 (95%)	704 (5%)	1924 (96%)	86 (4%)	3802 (95%)	198 (5%)	20,000
CMMD	678 (30%)	1574 (70%)	216 (29%)	530 (71%)	218 (29%)	528 (71%)	3744
INBreast	0 (0%)	0 (0%)	0 (0%)	0 (0%)	310 (76%)	100 (24%)	410

**Table 2:** AUC (±standard error) for each combination of training data and test data, for the various experimental treatments. Shaded are results where the training and test sets are from the same distribution.

		Test Dataset			
Training Dataset	Treatment	HMI	VinDR	CMMD	INBreast
HMI	Imbalanced	0.776 (±0.021)	0.700 (±0.032)	0.646 (±0.029)	0.818 (±0.033)
	Under-sampled	0.767 (±0.023)	0.664 (±0.027)	0.654 (±0.030)	0.803 (±0.040)
	Over-sampled	0.762 (±0.023)	0.699 (±0.028)	0.641 (±0.031)	0.789 (±0.039)
	Class Weighting	0.786 (±0.021)	0.650 (±0.030)	0.660 (±0.030)	0.795 (±0.037)
	Artifacted	**0.807** (±0.021)	**0.760** (±0.025)	**0.730** (±0.028)	**0.845** (±0.030)
VinDR	Imbalanced	0.622 (±0.026)	0.757 (±0.028)	0.671 (±0.027)	0.732 (±0.045)
	Under-sampled	0.630 (±0.026)	0.691 (±0.027)	0.680 (±0.030)	0.744 (±0.045)
	Over-sampled	0.646 (±0.028)	0.752 (±0.027)	0.668 (±0.030)	**0.824** (±0.038)
	Class Weighting	0.589 (±0.027)	0.739 (±0.030)	**0.685** (±0.029)	0.691 (±0.048)
	Artifacted	**0.686** (±0.025)	**0.768** (±0.027)	0.644 (±0.030)	0.799 (±0.039)
CMMD	Imbalanced	0.516 (±0.027)	0.520 (±0.032)	**0.727** (±0.026)	0.656 (±0.049)
	Under-sampled	0.559 (±0.028)	0.513 (±0.032)	0.690 (±0.028)	0.702 (±0.045)
	Over-sampled	**0.614** (±0.026)	**0.667** (±0.031)	0.719 (±0.028)	**0.711** (±0.048)
	Class Weighting	0.495 (±0.027)	0.541 (±0.027)	0.681 (±0.027)	0.624 (±0.048)

**Table 3:** Metrics (±standard error) at an operating point of 0.5, where the test set and training set come from the same distribution.

Training Dataset	Treatment	Sensitivity	Specificity	MCC
HMI	Imbalanced	0.574 (±0.033)	0.824 (±0.027)	0.418 (±0.042)
	Under-sampled	0.690 (±0.034)	0.709 (±0.029)	0.399 (±0.043)
	Over-sampled	0.595 (±0.033)	0.776 (±0.031)	0.379 (±0.037)
	Class Weighting	0.515 (±0.034)	**0.857** (±0.023)	0.398 (±0.044)
	Artifacted	**0.753** (±0.028)	0.689 (±0.031)	**0.443** (±0.043)
VinDR	Imbalanced	0.004 (±0.010)	1.000 (±0.000)	0.033 (±0.063)
	Under-sampled	0.400 (±0.048)	0.837 (±0.008)	0.141 (±0.032)
	Over-sampled	0.263 (±0.046)	**0.989** (±0.004)	**0.378** (±0.046)
	Class Weighting	**0.644** (±0.043)	0.708 (±0.011)	0.170 (±0.022)
	Artifacted	0.499 (±0.052)	0.873 (±0.009)	0.253 (±0.030)
CMMD	Imbalanced	0.781 (±0.027)	0.479 (±0.047)	**0.261** (±0.052)
	Under-sampled	0.336 (±0.028)	**0.894** (±0.029)	0.236 (±0.040)
	Over-sampled	**0.803** (±0.026)	0.433 (±0.047)	0.246 (±0.055)
	Class Weighting	0.520 (±0.030)	0.727 (±0.032)	0.245 (±0.041)

## Data Availability

Three of the datasets used in this study are publicly available: INBreast [17] is available on request; the VinDr-Mammo [11] dataset is available through PhysioNet at https://www.physionet.org/content/vindr-mammo/1.0.0/ (accessed on 15 November 2022); and CMMD [10] is available from the Cancer Imaging Archive at https://wiki.cancerimagingarchive.net/pages/viewpage.action?pageId=70230508 (accessed on 15 November 2022).
